# Lactate-Induced HBEGF Shedding and EGFR Activation: Paving the Way to a New Anticancer Therapeutic Opportunity

**DOI:** 10.3390/cells13181533

**Published:** 2024-09-13

**Authors:** Valentina Rossi, Alejandro Hochkoeppler, Marzia Govoni, Giuseppina Di Stefano

**Affiliations:** 1 Department of Medical and Surgical Sciences (DIMEC), Section of General Pathology, University of Bologna, 40126 Bologna, Italy; valentina.rossi78@unibo.it (V.R.); marzia.govoni@unibo.it (M.G.); 2 Department of Pharmacy and Biotechnology (FaBiT), University of Bologna, 40126 Bologna, Italy; a.hochkoeppler@unibo.it

**Keywords:** cancer cell metabolism, lactate, EGFR, HBEGF

## Abstract

Cancer cells can release EGF-like peptides, acquiring the capacity of autocrine stimulation via EGFR-mediated signaling. One of these peptides (HBEGF) was found to be released from a membrane-bound precursor protein and is critically implicated in the proliferative potential of cancer cells. We observed that the increased lactate levels characterizing neoplastic tissues can induce the release of uPA, a protease promoting HBEGF shedding. This effect led to EGFR activation and increased ERK1/2 phosphorylation. Since EGFR-mediated signaling potentiates glycolytic metabolism, this phenomenon can induce a self-sustaining deleterious loop, favoring tumor growth. A well characterized HBEGF inhibitor is CRM197, a single-site variant of diphtheria toxin. We observed that, when administered individually, CRM197 did not trigger evident antineoplastic effects. However, its association with a uPA inhibitor caused dampening of EGFR-mediated signaling and apoptosis induction. Overall, our study highlights that the increased glycolytic metabolism and lactate production can foster the activated state of EGFR receptor and suggests that the inhibition of EGFR-mediated signaling can be attempted by means of CRM197 administered with an appropriate protease inhibitor. This attempt could help in overcoming the problem of the acquired resistance to the conventionally used EGFR inhibitors.

## 1. Introduction

EGFR (also known as ErbB1/HER1) is one of the members of the ErbB receptor tyrosine kinase family [1]. In cancer cells, its aberrant activation is primarily mediated by gene amplification and gives rise to signaling cascades promoting cell proliferation and playing a key role in cell survival [2]. These findings suggest that EGFR hyperactivation can take an active part in the neoplastic change process and in conferring substantial growth and survival advantages to cancer cells. In addition, EGFR-mediated signaling was found to play a fundamental role in the initial steps of the epithelial/mesenchymal transition (EMT) [3,4], while treatment with EGFR inhibitors appeared in some contexts to induce changes in cell morphology, from a spindle shape to a more epithelial phenotype [5].

In light of these data, it is not surprising that, in patients with multiple cancer forms elevated EGFR expression was revealed to be a strong prognostic feature, usually associated with poor outlook and indicative of reduced recurrence-free or overall survival rates [6]. Conversely, the clinical use of EGFR inhibitors often appeared to produce substantial therapeutic benefits [7].

A common feature of rapidly proliferating cells is the increased level of glycolysis, and an activated EGFR pathway was also found to be one of the major regulators of cancer cell metabolism [8]. Stimulation of EGFR by its ligands is known to accelerate glucose consumption and foster the so-called Warburg effect, resulting in increased lactate production by cancer cells [9]. At the molecular level, this metabolic reprogramming was shown to be mediated through the concurrent upregulation of hexokinase-2 (HK2) expression and phosphorylation of pyruvate kinase M2 (PKM2), which alters the oligomerization state of the enzyme and inhibits its activity [10]. These changes cause at the same time an acceleration in the first step of glycolysis, together with a stall at the end of the glycolytic cascade [8]. As a result, an accumulation of metabolic intermediates is generated in cell cytoplasm, with lactate being the prevailing one.

In neoplastic tissues, released lactate levels were found to be up to 40-fold increased, compared to normal ones [11]. This metabolite can work as a signaling molecule: it increases the migratory potential of cancer cells and their metastatic spread [12] and, interestingly, it was found to further promote glycolytic metabolism by leading to increased MYC and LDH-A expression [13]. In recent years, studies performed by our research group have highlighted the potential of lactate to interfere with some mechanisms of action of antineoplastic treatments, leading to reduced drug response [14,15,16].

Taken together, these lactate-induced effects can be explained by its role in the regulation of gene expression. Initially, lactate was shown to affect gene expression through the inhibition of histone deacetylases [17]; more recently, Zhang et al. discovered a new epigenetic modification of histones’ lysine residues, “lactylation”, that directly stimulates gene transcription from chromatin [18]. For these properties, lactate has been proposed as a bridge linking the metabolic activation of cancer cells to their epigenetic changes [19].

Based on these premises, the aim of the present study was to investigate a possible role of lactate in fostering the activated state of the EGFR pathway. In particular, we first considered the observation that some cancer cells can directly release EGF-like peptides, acquiring the capacity of autocrine stimulation [20]. One of these peptides is the soluble form of heparin-binding EGF (HBEGF). Initially synthesized as a transmembrane precursor protein, HBEGF is cleaved at the cell surface by proteases and is released in a soluble form [21], a process usually referred to as “HBEGF shedding”. Released HBEGF was found to be a potent mitogen [22]; furthermore, it was found to be implicated in tumor progression and chemotherapy resistance [23,24].

To test the activation of EGFR by lactate, we used two human cell cultures, in which the expression of HBEGF was clearly documented in published studies: MDA-MB-231 [25] and HT-29 [26]. These cultures are representative of clinically relevant tumor forms: triple-negative breast cancer (MDA-MB-231) and colon adenocarcinoma (HT-29). We routinely maintained the two cultures in a medium containing a physiological glucose level, equivalent to that characterizing healthy human tissues. By exposing the cultures to a lactate level similar to that detected in the microenvironment of neoplastic tissues, we verified whether this metabolite could play a role in sustaining the HBEGF-mediated EGFR pathway.

## 2. Materials and Methods

### 2.1. Cell Cultures and Treatments

MDA-MB-231 and HT-29 cells (ATCC, Manassas, VA, USA) were grown in low-glucose (1 g/L) DMEM (31885-023, Thermo Fisher Sci., Waltham, MA, USA) supplemented with 100 U/mL penicillin/streptomycin (P0871, Merck, Darmstadt, Germany), 2 mM glutamine (G7513, Merck) and 10% FBS (F7524, Merck). L-lactate (L7022, Merck) was dissolved in culture medium at a 20 mM concentration. Before experiments, both cell cultures were adapted to grow in the lactate-containing medium for at least 3 months. This time interval was found to be necessary for the complete manifestation of the phenotype linked to the epigenetic effects of lactate. MDA-MB-231 cells were found to adequately proliferate in the lactate-containing culture medium without any sign of alteration. HT-29 culture showed delayed substrate adhesion in the presence of lactate; for this reason, in routine cell passages these cells were allowed to adhere for 16 h to the new substrate before adding lactate to the culture medium. Both cultures were routinely split once a week.

Cisplatin (HY-17394, MedChem Express, Monmouth Junction, NJ, USA) was dissolved in 0.9% NaCl. CRM197 (Cross Reacting Material 197) was obtained in lyophilized form (SC-203924, Santa Cruz Biotechnology, Dallas, TX, USA); it was dissolved in ultra-pure water (W4502, Merck) and stored at −80 °C. BC11 hydrobromide (4372, lot 1A/270652, Bio-Techne, Minneapolis, MN, USA) was dissolved in DMSO (C6164, Merck) (30 mM) and stored at −20 °C.

### 2.2. Real-Time PCR

Real-time PCR (RT–PCR) was first performed using MDA-MB-231 cells, to compare the gene expression of the lactate-exposed culture with that of the same culture routinely maintained in high-glucose (4 g/L) DMEM (D6546, Merck). This procedure helped in identifying a small group of genes specifically responsive to lactate, excluding the effects of upregulated glycolysis. Subsequently, the expression of these genes was also evaluated in lactate-exposed HT-29 cells. Exponentially growing cells from T25 flasks were used. RNA was extracted by using an RNA isolation kit (83913, Merck) and was quantified spectrophotometrically (ONDA Nano Genius photometer, OPTO-LAB Instruments, Modena, Italy). Retro-transcription to cDNA was performed by using the Revert Aid First Strand cDNA Synthesis Kit (K1691, lot 00291984, Thermo Fisher Sci.), in different steps: 5 min denaturation at 65 °C, 5 min annealing at 25 °C, 1 h retro-transcription at 42 °C and 5 min at 70 °C. RT–PCR analysis was performed by using 20 ng cDNA, Sso-Advanced Universal SYBR Green Supermix (1725271, lot 64545727, Bio-Rad, Hercules, CA, USA) and different primers mixtures. All the primers used for the PCR experiments were predesigned (KiCqStart^®^, Merck). Internal control genes were B2M, CYP33, RPS13 and TUBA. The list of oligonucleotide primer pairs is shown in Table 1. For all genes, the annealing temperature of primers was 60 °C and the thermal cycler (CFX96^TM^ Real Time System, Bio-Rad, Hercules, CA, USA) was programmed as follows: 30 s at 95 °C; 40 cycles of 15 s at 95 °C; 30 s at 60 °C. The data of RT–PCR experiments were analyzed by applying the 2^−CT^ method [27].

### 2.3. Immunoblotting Experiments

These experiments were performed in control and lactate-exposed MDA-MB-231 and HT-29 cells, to confirm the data obtained by RT–PCR and assess the activation state of EGFR. To evaluate the effects of HBEGF and uPA inhibitors in lactate-exposed MDA-MB-231 cells, they were exposed for 24 h to CRM197 (2 µg/mL, corresponding to 32 nM) and BC11 (100 µM).

Cultures (T-25 flasks, at 80% confluence) were harvested and lysed in 50 µL RIPA buffer containing protease and phosphatase inhibitors (cOmplete™, 04693116001, Merck; Halt™, 78420, Thermo Fisher Sci.). Then, 70 µg of protein (determined by using Bradford reagent, B6916, Merck) was loaded onto precast 4–12% polyacrylamide gels for electrophoresis (Bolt^TM^, 04120, Thermo Fisher Sci.) and run at 170 V. The separated proteins were blotted on low-fluorescent Hybond^TM^ PVDF membranes (10600060, lot A30730600, Cytiva Life Sciences, GE Healthcare, Chicago, IL, USA) using the Bolt^TM^ transfer system and maintaining 60 mA for 16 h. The blotted membranes were blocked with 5% BSA (A9418, Merck) in TBS-Tween and probed with the primary antibody. Actin was used as a loading control in all experiments. The primary and secondary antibodies used for the immunoblotting experiments are listed in Table 2.

### 2.4. ELISA for the Detection of HBEGF Released in Culture Medium

Both control and lactate-exposed cells were used; released HBEGF was assessed by applying a commercially available ELISA assay (EHHBEGF, lot 271111623, Thermo Fisher Sci.).

Cells (3 × 10^5^ MDA-MB-231 and 5 × 10^5^ HT-29) were seeded in each well of 6-well plates in 750 µL culture medium and maintained for 24 h in a humidified incubator at 37°. During this time, lactate-exposed cells were also treated with 100 µM BC11. At the end of treatment, culture medium was recovered, filtered through a 0.22 μm syringe filter and centrifuged for 30 min at 2000 RCF. Samples were then aliquoted and stored at −80 °C. The ELISA assay was performed by using 100 µL samples and following the manufacturer instructions. At the end of the procedure, absorbance of samples was evaluated at 450 nm, with the aid of a Synergy HT–BioTek plate reader (Agilent Technologies, Santa Clara, CA, USA). For both cell lines, ELISA procedure was replicated at least 4 times.

### 2.5. Cell Proliferation Experiments

To evaluate the effect on cell proliferation caused by the different cell culture media and by the used inhibitors, we adopted a procedure based on crystal violet (CV; C0775, Merck) staining. CV binds to nucleic acids and allows a precise estimate of cell number, which can be calculated with the aid of a calibration curve. Before each experiment, a plot reporting the CV absorbance values of scalar amounts of cells was obtained. These data were fitted by using the linear regression analysis; the resulting equation was used to calculate the number of cells at the beginning (T = 0) and at the end of experiments (T = 24 h).

Control and lactate-exposed MDA-MB-231 and HT-29 cells (1 × 10^4^ cells/well) were seeded in 96-multiwell plates and let to adhere overnight. Cultures were then exposed to treatments for 24 h. Cisplatin was administered at a dose of 50 µM; CRM197 at 32 nM; BC11 at the doses of 50, 75 and 100 µM. At the end of treatment, medium was removed and the cells were fixed with 1% glutaraldehyde (G6257, Merck) for 20 min. After staining with CV (0.01% in distilled water, 30 min) wells were washed with PBS (14190, Thermo Fisher Sci.) and CV was solubilized by shaking in 70% ice-cold ethanol for 30 min at room temperature. Absorbance was evaluated at 570 nm by using the Multiskan EX plate reader (Thermo Fisher Sci.). In each experiment and for each treatment, the increase (or decrease) in cell number during the 24 h was calculated.

### 2.6. Assay of Lactate Levels

Control and lactate-exposed MDA-MB-231 cells were seeded in triplicate in 24-well plates (2 × 10^5^ cells/well) and let to adhere. They were then treated with 32 nM CRM197 and 100 µM BC11, given individually or in combination for 16 h. Culture medium was then replaced with Krebs–Ringer buffer (300 µL/well). The concentration of lactate released in Krebs–Ringer buffer was evaluated after 5 h of incubation at 37 °C, following the procedure detailed in [16].

### 2.7. Wound Healing Assay

For this experiment, control and lactate-exposed MDA-MB-231 cells were used. Lactate-exposed cells were also treated with the association of CRM197 (32 nM) and BC11 (100 µM). Cells were seeded in triplicate in 6-well plates (1.5 × 10^6^ cells/well) and cultured until they had reached 100% confluence.

Artificial wounds were then created using a 10 µL pipette tip. The detached cells were washed away with PBS and cultures were exposed to a medium supplemented with 2% FBS. The wound areas were captured with an inverted microscope (Primovert, Carl Zeiss Microscopy, Gottingen, Germany) at 0, 6, 20, 24 and 30 h and their repopulation was analyzed by using the ImageJ software, using the Wound Healing Size Tool according to the procedure described in [28]. A similar experiment was performed on HT-29 cells; in this culture, lactate-exposed cells were treated with the single BC11 treatment (100 µM).

### 2.8. Clonogenicity Assay

Control and lactate-exposed MDA-MB-231 cells were seeded in duplicate in 6-well plates (5 × 10^2^ cells/well) and let to adhere overnight. Lactate-exposed cells were also treated with the association of CRM197 (32 nM) and BC11 (10 µM). Cell colonies became clearly evident after 8 days; at this time, they were fixed and stained with 6% glutaraldehyde and 0.5% CV in PBS (30 min at room temperature). Stained colonies were dissolved with 10% SDS (400–800 µL). The absorbance of the solutions was measured with the aid of the Multiskan EX plate reader at 570 nm.

A similar experiment was also performed on HT-29 cultures. In this case, lactate-exposed cells were treated only with BC11, given at 10 and 20 µM; colonies became evident after 15 days.

### 2.9. E-Cadherin Immunostaining

Lactate-exposed MDA-MB-231 cells were seeded on glass slides posed in the wells of a 6-well plate (1500 cells/well). They were allowed to adhere and were then treated with 32 nM CRM197 for 8 days, with medium renewal at day 4. After this time, cells were fixed with 4% paraformaldehyde (76240, Merck) and permeabilized with 70% ethanol. Glass slides were then treated with a blocking solution containing 5% BSA and exposed to a mouse monoclonal anti-human E-cadherin antibody (see Table 2) (1 ng/mL in BSA 5%, 16 h, at 4 °C). Binding was revealed with the aid of a FITC-conjugated anti-mouse polyvalent-(G, A, M)-immunoglobulins, produced in goat (see Table 2) (1:400 in BSA 5%, 30 min at room temperature). Glass slides were then mounted with a solution of DAPI (2 g/mL) and DABCO. Pictures of cells were taken at a 600× magnification, using a Nikon Eclipse-E600 epifluorescence microscope (Nikon Corporation, Tokyo, JPN) equipped with filters for FITC (excitation: 495 nm; emission: 519 nm) and DAPI (excitation: 350 nm; emission: 460 nm) and with a DXM1200F Nikon digital camera (ACT-2U software, version 1.21.41.176; camera gain: 12; camera offset: default).

### 2.10. Statistical Analysis

Results were obtained from at least two independent experiments, performed with triplicate samples. Data were analyzed using the GraphPad Prism 5 software. For each experiment, the adopted statistical evaluation is described in the corresponding paragraph of the Results section. Data were expressed as mean values ± SE and were calculated using all the results obtained from the independent experiments; the significance level was set at *p* < 0.05.

## 3. Results

### 3.1. Lactate Upregulates Urokinase-Type Plasminogen Activator (uPA), Leading to HBEGF Shedding

To highlight the potential role of lactate in EGFR activation, in the first set of experiments we adopted for MDA-MB-231 cells three different culture conditions: (a) maintenance in the presence of physiologic glucose levels (1 g/L, Low-Glc DMEM, control cells); (b) maintenance in Low-Glc DMEM supplemented with 20 mM lactate (lactate-exposed cells); (c) maintenance in a conventional, high-glucose (4 g/L) DMEM (High-Glc DMEM). The lactate supplementation used in medium (b) matches the level of metabolite detected in the microenvironment of neoplastic tissues [29,30,31] and in previous studies was found to cause a significantly increased level of histone-3 acetylation [15]. As specified in Section 2.1, MDA-MB-231 cells were grown in the above-described conditions for at least three months before beginning the experiments. The same phase of adaptation was adopted for HT-29 cultures, which were maintained only in Low-Glc DMEM with or without the 20 mM lactate supplementation.

**Figure 1:**
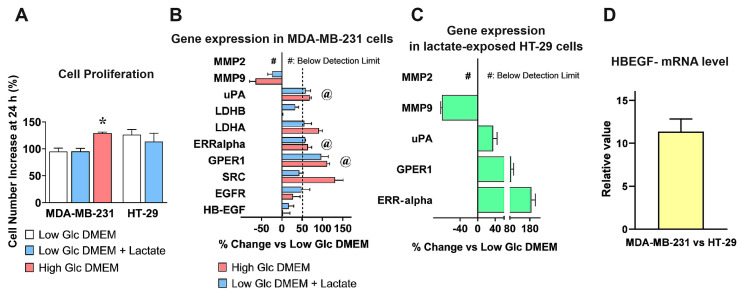
mRNA levels of EGFR and of proteins involved in its activation, assessed by RT–PCR. (**A**) A preliminary evaluation was carried out to verify whether the different DMEM formulations could affect the proliferation dynamics of cell cultures. *, a statistically significant difference with *p* < 0.05 was found in cultures maintained in High-Glc DMEM vs those maintained in the Low-Glc medium (ANOVA followed by Tukey’s post-test). (**B**) mRNA levels in MDA-MB-231 cells grown in Low-Glc DMEM were compared to those detected in High-Glc DMEM maintained cells and in cells exposed to Low-Glc DMEM + lactate. For genes’ selection, a threshold at ≥50% increase was set (dotted line). Furthermore, only genes showing no statistically significant difference between High-Glc DMEM grown cells and those exposed to Low-Glc DMEM + lactate were considered. These criteria were met by uPA, ERR-alpha, and GPER1 (@). The selected genes (@) were also studied in HT-29 cells, cultured in Low-Glc DMEM and exposed to lactate (**C**), together with MMP9 and MMP2 (two proteases involved in HBEGF shedding). In these cells, RT–PCR analysis substantially confirmed the data obtained in MDA-MB-231 cultures. The statistical evaluations applied to the data shown in (**B**,**C**) are detailed in the text. (**D**) Comparison of HBEGF mRNA levels between the two cell cultures.

As shown in Figure 1A, lactate supplementation in Low-Glc DMEM did not affect the proliferation of MDA-MB-231 cells, and both cultures maintained in the Low-Glc medium almost doubled their cell number in 24 h. On the contrary, a significantly increased cell proliferation (equal to about 35%) was observed in the culture maintained in High-Glc DMEM. A similar experiment performed in the HT-29 culture showed again that lactate supplementation in the Low-Glc medium did not modify cell proliferative potential.

We then analyzed by RT–PCR the mRNA level of EGFR and of a small number of proteins involved in its activation and/or having prognostic value in cancer diseases: MMP-2 and -9 [32,33]; uPA [34,35,36]; G Protein Coupled Receptor 1 (GPER1) [37,38,39]; Estrogen Related Receptor alpha (ERR-alpha) [39,40,41]; SRC [42]; LDH-A and -B [43]; HBEGF. The adopted culture conditions helped us in discriminating between the effects specifically ascribable to lactate and those caused by an overactivated glycolytic flux (High-Glc DMEM).

The obtained results are shown in Figure 1B. The genes considered for further experiments were those showing: (1) a ≥ 50%-increased level and (2) a similar expression in High-Glc DMEM grown and in lactate-exposed cells; differences were statistically evaluated by ANOVA followed by Tukey’s post-test. The selected conditions were met by uPA (Low-Glc DMEM vs. +Lactate, *p* < 0.05; Low-Glc DMEM vs. High-Glc DMEM, *p* < 0.01; High-Glc DMEM vs. Low-Glc DMEM+Lactate, NS), GPER1 and ERR-alpha (for both genes, Low-Glc DMEM vs. +Lactate, and vs. High-Glc DMEM, *p* < 0.01; High-Glc DMEM vs Low-Glc DMEM+Lactate, NS). Graph 1B also shows increased expression of LDH-A (+55% in lactate-exposed cells and +90% in High-Glc DMEM grown cells); this result was not considered, since the difference observed between the two culture media suggested a major contribution from enhanced glycolysis rather than from lactate alone. For the same reason, we did not consider the strongly increased level (+130%) of SRC, a tyrosine kinase engaged in breast cancer development and progression [42], which was observed only in cells exposed to High-Glc DMEM.

Media containing enhanced glucose levels are routinely used in research laboratories to maintain cell cultures and hasten their proliferation; our results suggest caution in following this procedure, since it showed the potential of changing gene expression, affecting the experimental results.

**Figure 2:**
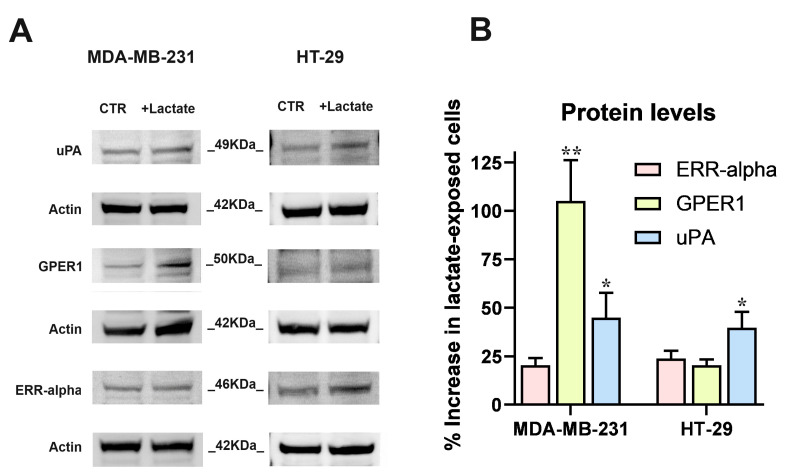
The results of RT–PCR experiments (Figure 1) were validated by the immunoblotting detection of proteins. (**A**) Images of the protein bands and of the used internal standard (Actin). (**B**) The densitometric reading of bands, normalized on Actin levels, was used to calculate the % increase of protein levels in lactate-exposed cells vs. control cultures, maintained in Low-Glc DMEM. Because of their limited extent, the increases of ERR-alpha (in both cell lines) and GPER1 (in HT-29 cultures) were not further considered. The results concerning uPA (in both cell cultures) and GPER1 (only in MDA-MB-231 cells) were analyzed by one-sample *t*-tests; * and ** indicate a statistically significant increase compared to the control cultures, with *p* < 0.05 and 0.01, respectively.

Interestingly, MMP-9 (a protease usually described as a potential HBEGF activator [33]) was significantly reduced as a consequence of lactate exposure, while MMP-2 expression was not detected in MDA-MB-231 cells. These results were confirmed in HT-29 cultures, adapted to grow in Low-Glc DMEM + 20 mM lactate for a period ≥3 months (Figure 1C). In these cells, lactate appeared to exert a much stronger effect on ERR-alpha expression. RT–PCR data obtained in HT-29 cells were evaluated by ANOVA followed by Dunnet’s post-test. A highly significant statistical difference was observed for GPER1 and ERR-alpha (*p* < 0.01 and 0.001, respectively). In this experiment, the increase of uPA mRNA did not reach the level of statistical significance (which, however, was observed in the immunoblotting detection of Figure 2B). It is noteworthy that, when the expression of HBEGF in the two cultures was compared, MDA-MB-231 showed a more than 10-times higher mRNA level compared to HT-29, suggesting a far lower dependence of these cells on HBEGF-mediated signaling (Figure 1D).

In both cell cultures, the RT–PCR data were validated by the immunoblotting evaluation of uPA, GPER1 and ERR-alpha shown in Figure 2. In this experiment, the strong increase of ERR-alpha detected in HT-29 cells was not confirmed and this protein appeared to be scarcely upregulated in both the cell cultures; GPER1 protein level was markedly increased in lactate-exposed MDA-MB-231 cells and uPA showed a statistically significant increase, equal to about 40%, in both lactate-exposed cultures.

In both normal and malignant breast cells, GPER1 was found to be involved in HBEGF-mediated EGFR pathway activation [37]; furthermore, endogenously produced uPA was proposed as a major determinant leading to ERK1/2 phosphorylation in MDA-MB-231 cells [44]. Although HBEGF expression was not significantly upregulated as a result of lactate exposure, following the above-mentioned results, we verified whether lactate-exposed MDA-MB-231 and HT-29 cells had acquired the potential of releasing higher levels of HBEGF in medium, through a uPA-mediated mechanism. To this aim, we used BC11, a specific uPA inhibitor [45].

**Figure 3:**
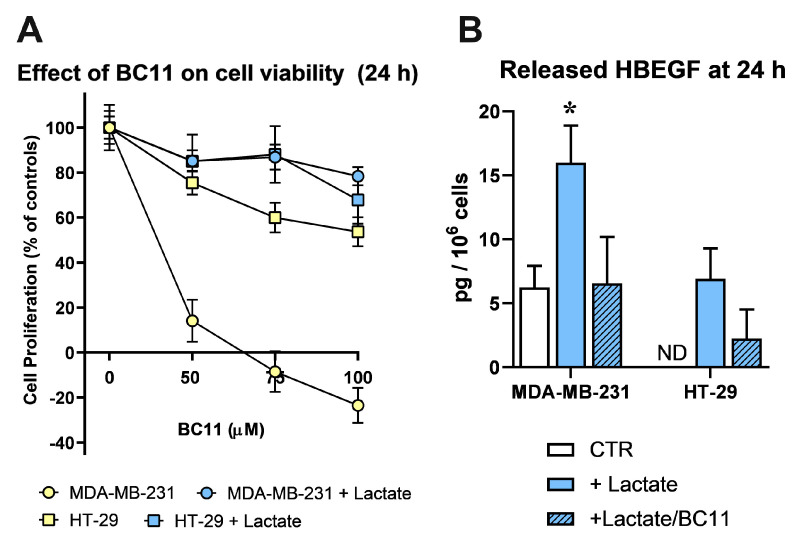
(**A**) Effects caused by BC11 (a uPA inhibitor) on the proliferation of control (grown in Low-Glc DMEM) and lactate-exposed MDA-MB-231 and HT-29 cells, at 24 h. Lactate was found to drastically reduce the toxic effects of BC11 in MDA-MB-231 culture. No significant difference was observed between the two lactate-exposed cultures at all the tested doses of BC11. (**B**) Detection of released HBEGF (24 h) in control (Low-Glc DMEM) cultures and in cells exposed to lactate or lactate + BC11 (100 µM). In MDA-MB-231 cultures, lactate significantly increased HBEGF shedding (*, *p* < 0.05, assessed by ANOVA followed by Dunnett’s post-test). In control HT-29 cells, released HBEGF was undetectable (ND), but reached the limit of detectability in lactate-exposed cells. In both lactate-exposed cultures, BC11 supplementation reduced the level of released HBEGF and no statistically significant difference was observed between control cells and cells exposed to lactate/BC11.

Since BC11 was shown to exert toxic effects on MDA-MB-231 cells [45], in a preliminary experiment we evaluated the tolerability of this compound on the lactate-exposed cultures; results are shown in Figure 3A.

In agreement with published data, BC11 severely affected MDA-MB-231 cell viability in a dose-dependent manner, while HT-29 cells appeared to better tolerate this inhibitor. Interestingly, in lactate-exposed MDA-MB-231 cells, the effect of BC11 was drastically reduced and the two lactate-exposed cultures were shown to tolerate BC11 similarly, up to 100 µM, 24 h. These conditions were hence adopted to evaluate released HBEGF in medium.

Figure 3B shows the results obtained using a commercially available ELISA, specifically designed for the quantification of HBEGF in its soluble form. In lactate-exposed MDA-MB-231 cultures the level of soluble HBEGF appeared to be 3-fold increased, compared to control cells, maintained in Low-Glc DMEM; BC11 supplementation almost completely inhibited the effect of lactate supplementation. As expected, in control HT-29 cultures, soluble HBEGF was undetectable; it reached the limit of detectability in lactate-exposed cells and, also in this culture, BC11 supplementation was found to reduce the release of the soluble form.

These results clearly demonstrated that the upregulated expression of uPA observed in lactate-exposed cells can play a role in releasing the soluble form of HBEGF.

### 3.2. Lactate-Exposed Cells Show Signatures of Activated EGFR Pathway and Reduced Response to Cisplatin

To verify whether the uPA-increased HBEGF shedding can result in enhanced activation of EGFR-mediated signaling, we assessed the phosphorylation level of the receptor and of its downstream kinases (ERK1/2) by applying an immunoblotting assay.

**Figure 4:**
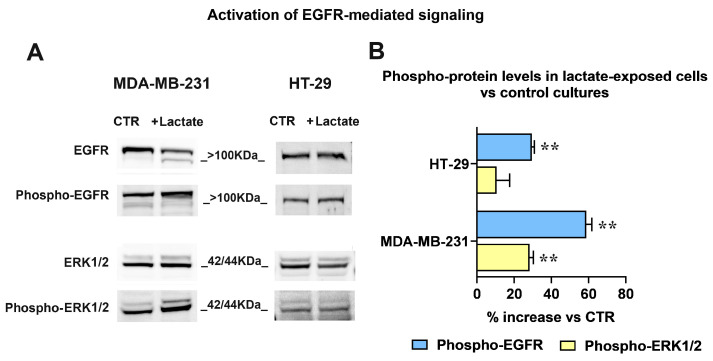
(**A**) Immunoblotting detection of EGFR and ERK1/2 phosphorylation. The signal intensity ratios (phospho-protein/protein) were calculated and the obtained values were used to assess the % increase in phospho-EGFR and phospho-ERK1/2 observed in lactate-exposed cells (**B**). For both phospho-EGFR/EGFR and phospho-ERK1/2/ERK1/2 immunoblotting analyses, the same sample was used and was run in parallel experiments; gels and blots were processed in parallel. The data shown in (**B**) were statistically evaluated by one-sample *t*-tests. In both cell cultures the increased phosphorylation of EGFR reached the level of statistical significance; on the contrary, phospho-ERK1/2 was significantly increased only in MDA-MB-231 cells. **, *p* < 0.01.

The obtained results are shown in Figure 4. The immunoblotting detection of EGFR (Figure 4A) revealed in lactate-exposed MDA-MB-231 cells two bands at a >100 kDa MW level; as previously described [46], these bands are diagnostic of the glycosylated forms of the receptor and indicate the presence of activated EGFR. The higher activated state was also confirmed by the increased level of phospho-EGFR (Tyr1068) and of phospho-ERK1/2 (Thr202/Tyr204) observed in lactate-exposed cells, compared to the control culture.

In lactate-exposed HT-29 cells, the EGFR signal appeared as a single band; however, the immunoblotting evaluation showed enhanced phosphorylation levels of the receptor also in these cells.

For both cultures, the bands’ densitometric ratios phospho-EGFR/EGFR and phospho-ERK1/2/ERK1/2 were calculated and are plotted in the bar graph of Figure 4B. The densitometric analysis evidenced a difference between the two cell lines: compared to their respective control cultures, in lactate-exposed MDA-MB-231 cells a significantly higher phosphorylation level was observed in both EGFR and ERK1/2. On the contrary, in lactate-exposed HT-29 cells the phosphorylation of the ERK1/2 downstream kinases was not significantly increased.

**Figure 5:**
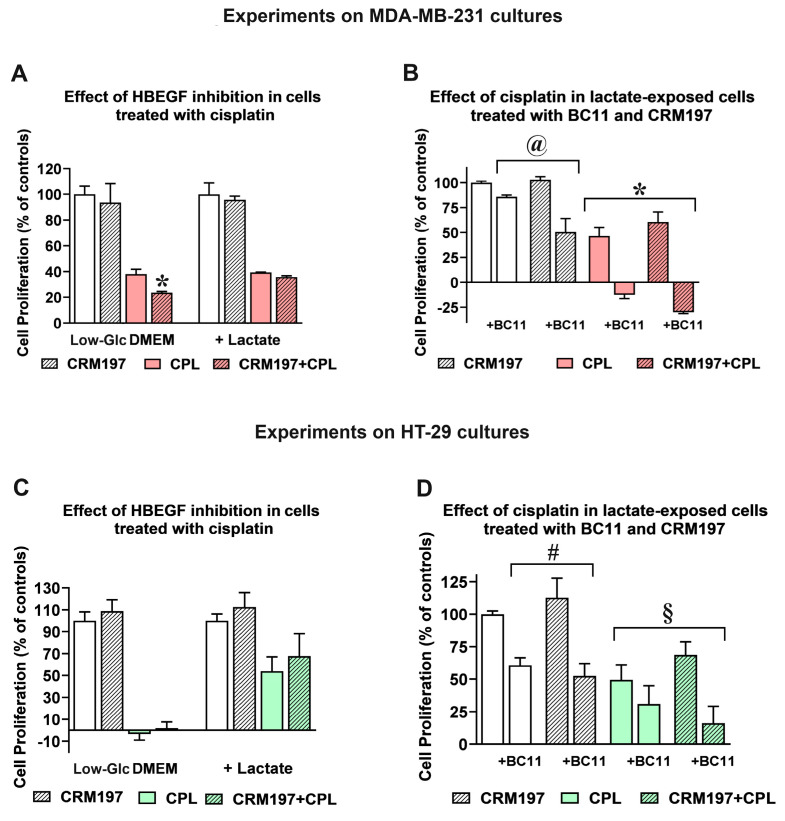
Effect of cisplatin (CPL) in control and lactate-exposed cultures. (**A**) In MDA-MB-231 cells grown in Low-Glc DMEM, the antiproliferative effect of 50 µM CPL was increased by CRM197, given at 32 nM (* *p* < 0.05, as assessed by *t*-test). This effect was not observed in lactate-exposed cells. (**B**) Lactate-exposed cells were exposed to CPL to evaluate the effect of CRM197 and BC11 on the drug response. Data were analyzed by ANOVA, followed by Tukey’s post-test. @: a statistically significant difference was observed between cell samples treated with BC11 and those exposed to BC11+CRM197 (*p* < 0.05). *: BC11 significantly increased the effect of CPL (*p* < 0.001). (**C**) In HT-29 cells grown in Low-Glc DMEM, the antiproliferative effect of 50 µM CPL was not modified by CRM197. Lactate-exposed cells showed a reduced response to CPL and, again, this effect was not modified by CRM197. (**D**) The experiments shown in (**B**) were replicated in HT-29 cultures. #: no statistically significant difference was observed between cell samples treated with BC11 and those exposed to BC11 + CRM197. §: the increased antiproliferative effect observed in cell samples exposed to CPL/CRM197/BC11 did not reach the level of statistical significance, when compared to the single CPL treatment. In these experiments, no difference in the proliferation rate was observed between cells maintained in Low Glc DMEM and those cultured in Low Glc DMEM + lactate.

Released HBEGF and the consequent EGFR pathway activation were repeatedly shown to be associated with chemoresistance [24], while EGFR inhibition showed the potential of improving the effects of chemotherapy and radiation therapy [47]. For this reason, in following experiments we evaluated the response of the control and lactate-exposed cultures to cisplatin, a chemotherapeutic agent currently used in the treatment of several neoplastic conditions [48]. In order to define the impact of uPA-induced HBEGF shedding on the antineoplastic action of cisplatin, in these experiments we also used BC11 and CRM197, a well-characterized HBEGF inhibitor [49]. Both cell lines were exposed to 50 µM cisplatin for 24 h; the obtained results are shown in Figure 5.

In MDA-MB-231 cultures (Figure 5A), CRM197 (2 µg/mL, corresponding to 32 nM) did not affect the proliferation of both control and lactate-exposed cells, and the single administration of cisplatin produced in the two cell cultures superimposable effects. When the two compounds were administered in combination, a statistically significant contribution of CRM197 in increasing the effect of cisplatin was observed only in cells maintained in Low-Glc DMEM, suggesting for this culture higher susceptibility to EGFR pathway inhibition, with a consequently improved drug response [50].

A similar experiment was performed only on the lactate-exposed MDA-MB-231 culture to assess the effects of BC11 supplementation on the combined cisplatin/CRM197 treatment. Results are shown in Figure 5B. When administered in combination with cisplatin, 100 µM BC11 markedly increased the effect of this antineoplastic agent, causing evident cell death (a reduced cell number at 24 h, compared to that measured at the beginning of treatment); this antineoplastic effect was further increased by the CRM197 co-administration. Interestingly, the experiment on lactate-exposed cells highlighted a statistically significant difference between the effect of the combination CRM197/BC11 compared to that caused by the single CRM197 treatment; this result was considered for further experiments (see following paragraphs).

Figure 5C,D show the same experiments, replicated on HT-29 cultures. In the culture maintained in Low-Glc DMEM, 50 µM cisplatin significantly impacted on cell proliferation, and a further contribution given by the CRM197 supplementation was not observed. Interestingly, cisplatin susceptibility of lactate-exposed cells appeared to be evidently reduced; this finding is in complete agreement with previous results obtained by our research group, showing that in a colon adenocarcinoma cell line lactate supplementation significantly reduced the DNA damage signatures caused by this drug [14]. However, also in lactate-exposed HT-29 cells CRM197 supplementation did not affect the antineoplastic action of cisplatin. When the effect of BC11 was examined in lactate-exposed HT-29 cells (Figure 5D), this inhibitor did not succeed in increasing cisplatin activity; an improved effect was only observed with the triple combination cisplatin/CRM197/BC11, which, however, did not reach the level of statistically significance, when compared with the single cisplatin treatment.

Taken together, the less marked effects observed in the experiments performed in HT-29 cultures can be easily explained by the lower dependence of these cells on HBEGF-mediated signaling (Figure 1D). For this reason, further studies aimed at characterizing the effect of the CRM197/BC11 combination were performed in lactate-exposed MDA-MB-231 cells; some additional data have also been obtained in the HT-29 culture and have been reported in the Supplementary Materials fold.

### 3.3. The Combined Inhibition of HBEGF Shedding and Function Shows Antineoplastic Potential in MDA-MB-231 Cultures

**Figure 6:**
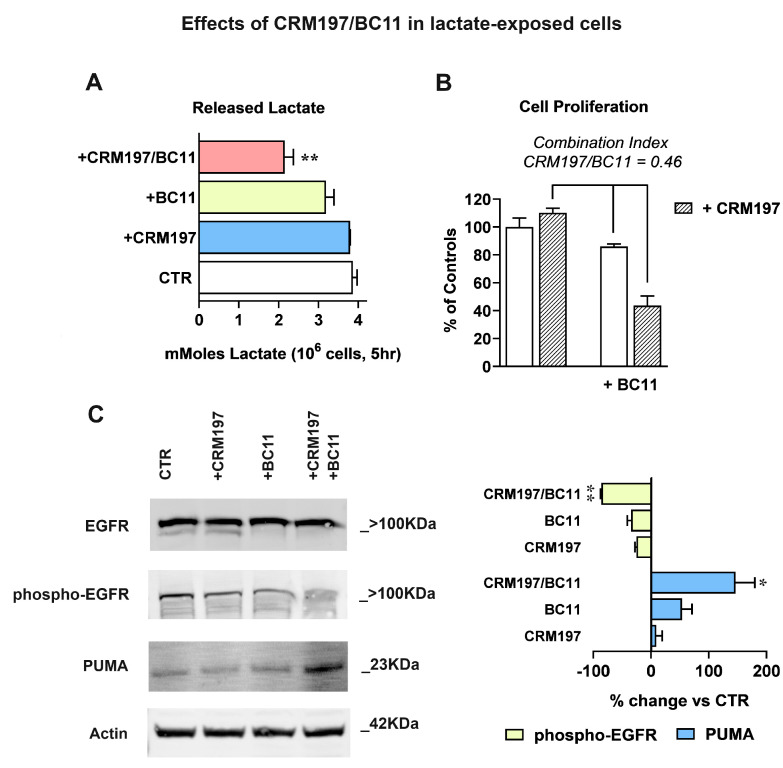
(**A**) Glycolysis inhibition, assessed by quantifying the released lactate. Data were analyzed by ANOVA followed by Dunnett’s post-test; a statistically significant reduction of lactate release was observed in cell samples exposed to the combined CRM197/BC11 treatment, with *p* < 0.01. (**B**) According to the method described in [51], the antiproliferative effect caused by CRM197/BC11 suggests synergism by the two compounds. (**C**) Immunoblotting evaluation of EGFR-mediated signaling shutdown and of apoptosis induction (PUMA). Phospho-EGFR band intensities were normalized on the corresponding EGFR signal; for this immunoblotting analysis the same sample was used and was run in parallel experiments; gels and blots were processed in parallel. PUMA band intensities were normalized on the corresponding Actin level. The bar graph shows the effects caused by the two compounds, given individually or in association. Data were analyzed by one-sample t-tests. The combination CRM197/BC11 significantly reduced phospho-EGFR, which became barely detectable, and markedly increased the level of PUMA. * and ** indicate a statistically significant difference compared to the control cultures, with *p* < 0.05 and 0.01, respectively.

Taking into account the results of Figure 5B, a further investigation on the effects of the CRM197/BC11 association was performed in lactate-exposed MDA-MB-231 cells (Figure 6). The compounds were always used at the concentrations tested in the previous experiments (32 nM CRM197 and 100 µM BC11). With the experiment of Figure 6A, we evaluated the effect of the two compounds on glycolytic metabolism, assessed by dosing the released lactate. For this experiment, cell cultures were exposed to the two compounds given individually or in association in their routinely used medium for 16 h; after this time, they were maintained in Krebs-Ringer buffer (a lactate-devoid medium allowing glycolysis) for additional 5 h, to assess the level of the released metabolite. As shown in the bar graph of Figure 6A, only the combined treatment CRM197/BC11 caused a statistically significant reduction of lactate release, suggesting impairment of glycolytic metabolism. Since activated glycolytic metabolism is needed to sustain cell proliferation and is associated with poor drug response of cancer cells [50], this effect can be hypothesized to contribute to the strongly increased cisplatin effect observed in lactate-exposed cells exposed to CRM197/BC11 (Figure 5B).

In the experiments of Figure 6B, we replicated the assay of cell proliferation by only including CRM197, BC11 and their combination. Again, the compounds’ association caused a statistically significant effect when compared to both CRM197 and BC11 given as single treatments. To better evaluate the power of the CRM197/BC11 combination, we applied to the obtained results the procedure described in [51], useful to assess synergism between pharmacologically active compounds. According to this procedure, synergism is suggested when the estimate of combination index [cells surviving to CRM197 + BC11/ (cells surviving to CRM197 × cells surviving to BC11)] returns a value < 0.8. As reported in Figure 6B, when applied to our experimental data this procedure gave a combination index = 0.46, suggesting a strong advantage given by the compounds’ association.

The enhanced power of CRM197/BC11 was also confirmed by the experiments in Figure 6C, which shows EGFR activation and apoptosis, assessed by immunoblotting. Bands’ densitometric evaluation is shown in the bar graph. In the untreated and in CRM197-exposed cells, the immunoblotting detection of EGFR showed again the presence of the two bands (see Figure 4) compatible with the activated receptor state, a feature which appeared to be suppressed by BC11. Accordingly, the detection of EGFR-phospho-Tyr1068 was more evidently reduced by BC11 and became barely detectable in cultures exposed to the combined treatment. An exactly opposite pattern was observed for the detection of the P53-Upregulated Mediator of Apoptosis (PUMA), which was not significantly affected by CRM197, was moderately increased by BC11, and appeared to be markedly enhanced following the CRM197/BC11 treatment.

Overall, the obtained results indicate for the CRM197/BC11 combination the potential of leading to a complete inhibition of the EGFR pathway, reproducing the effects of the receptor inhibitors used in clinical practice [47]. Therefore, our data suggest that the administration of CRM197/BC11 could not only empower the efficacy of a chemotherapeutic agent, such as cisplatin, but might also exhibit antineoplastic effects by itself.

### 3.4. Effects of the CRM197/BC11 Association on Infiltrative Growth and Cell Clonogenicity

The antineoplastic potential of CRM197/BC11 was finally characterized by examining the efficacy of this compound’s association in reducing the infiltrative growth and the clonogenic potential of the treated cells. Similar experiments were also performed on HT-29 cultures.

**Figure 7:**
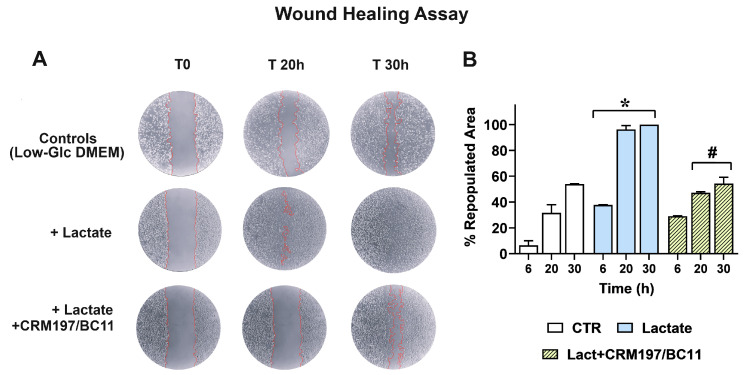
(**A**) Representative pictures of MDA-MB-231 cultures. The limits of the wound area have been outlined in red; repopulation was evaluated with the aid of the ImageJ software, as described in Section 2.7, and the percentage of healed wound over time is reported in (**B**). Data were analyzed by ANOVA followed by Tukey’s post-test. *: a statistically significant difference was observed between control and lactate-exposed cultures at all the considered time intervals (*p* < 0.01). #: CRM197/BC11 significantly reduced the effects of lactate at 20 and 30 h (*p* < 0.01).

Infiltrative growth was studied by applying a wound-healing assay; the results obtained in MDA-MB-231 cultures are shown in Figure 7. Figure 7A shows representative pictures of the cell cultures taken at 0, 20 and 30 h after wounding.

Compared to the control cells maintained in Low-Glc DMEM, lactate-exposed MDA-MB-231 cells exhibited higher infiltrative growth potential, since they were able to almost completely repopulate the wound area after 20 h; this effect appeared to be inhibited by CRM197/BC11. Panel B shows a quantitative estimate of the repopulated area in the three cultures, which also includes the first performed evaluation (6 h). A statistically significant difference was observed between lactate-exposed cells and their parental culture maintained in Low-Glc DMEM at all the considered time intervals (6, 20 and 30 h); the effect caused by CRM197/BC11 in lactate-exposed cells reached the level of statistical significance starting from 20 h after wounding. A video realized with the cultures’ images captured from 0 to 30 h has been included in the Supplementary Materials folder (Video S1).

Because of the reduced dependence on HBGF-mediated signaling shown by HT-29 cells, in this culture a similar experiment was performed by administering to control and lactate-exposed cells the single BC11 treatment, to assess whether the lactate-induced upregulation of uPA could play a role in increasing cell migration.

A picture showing the obtained results has been included in the Supplementary Materials folder (Figure S1). In spite of the well-documented role of uPA in facilitating the invasive behavior of cancer cells [34,52], no evidence of significantly increased repopulation of the wound area was observed in lactate-exposed HT-29 cells and, consequently, the administration of BC11 did not change the experimental outcomes. In our opinion, a possible explanation can be found in the compromised substrate adhesion shown by HT-29 cells when they are exposed to 20 mM lactate (see Section 2.1).

**Figure 8:**
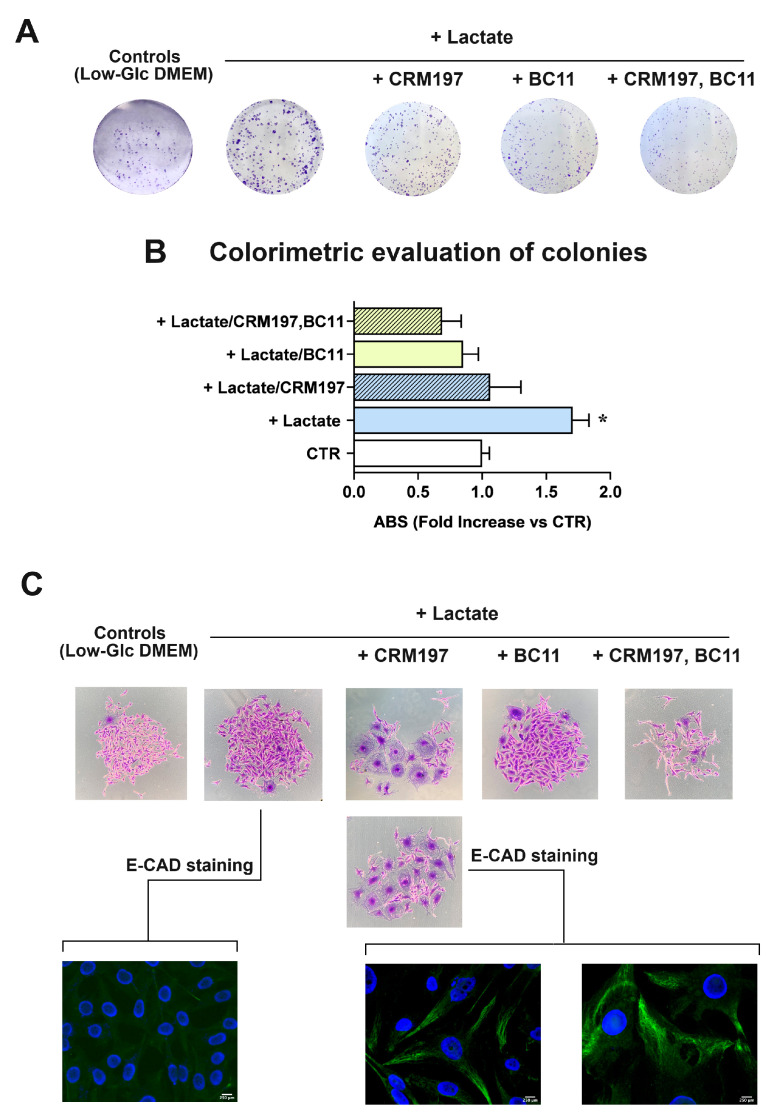
(**A**) Representative pictures of colonies formed by MDA-MB-231 cells, stained with CV. (**B**) Colorimetric evaluation of colonies. Data were evaluated by ANOVA, followed by Tukey’s post-test. *: a statistically significant difference was observed between lactate-exposed cells compared to controls and to all the applied treatments, with *p* values < 0.01–0.001. (**C**) High magnification pictures of colonies, showing the morphology changes induced by CRM197 (60×) and the immunostaining of E-cadherin (E-CAD) (600×). The green fluorescence indicative of E-CAD positive cells was clearly evident only in cells exposed to CRM197.

A conclusive experiment was aimed at evaluating the effect of CRM197/BC11 on the clonogenic potential of lactate-exposed MDA-MB-231 cells. In this long-lasting experiment, BC11 concentration was lowered to 10 µM to reduce the toxicity risk; the obtained results are shown in Figure 8. In this culture, new colonies were clearly evident after 8 days of incubation (Figure 8A); their number and extension were evaluated by colorimetry, after CV staining (Figure 8B).

In lactate-exposed cells, a statistically significant 1.7-fold increased clonogenic power was observed, when compared to the parental culture maintained in Low-Glc DMEM. When administered as individual treatments, both CRM197 and BC11 appeared to significantly reduce the clonogenic power of lactate-exposed cells, which became similar to that observed in Low-Glc DMEM cultured cells. A further reduction was caused by the compounds’ association: this treatment appeared to decrease cell clonogenicity to a level even lower than that observed in Low-Glc DMEM cultured cells. Among all the performed experiments, the clonogenicity assay was the one requiring long-lasting treatments (8 days). This longer exposure to CRM197 and BC11 allowed us to observe changes in MDA-MB-231 cell morphology, which are disclosed in Figure 8C. Pictures of Low-Glc DMEM cultured cells and of the untreated lactate-exposed cells show the typical morphology of the MDA-MB-231 culture, mainly characterized by spindle cells but also including a subpopulation of cells with enlarged cytoplasm [53,54]. In both pictures shown, one of these enlarged cells can be easily identified. The same pictures also show that, though maintaining the typical spindle morphology, lactate-exposed cells exhibit increased dimensions, probably as a consequence of the lactate-induced changes in gene expression.

Interestingly, treatment with CRM197 was associated with prominent changes in cell morphology, leading to the prevalence of the subpopulation characterized by the enlarged cytoplasm. According to previous studies [54], the two different morphologies of MDA-MB-231 cells are expression of cell subpopulations with different biological properties, with the spindle mesenchymal-like cells exhibiting higher activated glycolysis metabolism and metastatic potential, and the enlarged epithelial-like cells showing oxidative metabolism. Interestingly, these cell phenotypes were found to be plastically modulated by changes in cell energy metabolism [54]. Based on these observations and according to our results, it can be hypothesized that, although lacking direct antineoplastic power, CRM197 treatment could induce a mesenchymal–epithelial transition which should lead to the prevalence of cells with reduced metastatic potential. This hypothesis was confirmed by the E-cadherin (E-CAD) immunostaining shown in Figure 8C: in lactate-exposed cells, no evidence of E-CAD staining was observed, while E-CAD staining became clearly evident after the CRM197 treatment.

Figure 8C also shows that BC11 did not significantly affect MDA-MB-231 cell morphology and that the residual colonies observed following the combined CRM197/BC11 treatment appeared to be smaller and characterized by shrunk cells.

The clonogenicity assay was also performed in HT-29 cultures exposed to the single BC11 treatment, given at 10 and 20 µM; in this case, evident generation of colonies required about 15 days. The obtained results have been included in the Supplementary Materials folder (Figure S2). Compared to the MDA-MB-231 culture, in lactate-exposed HT-29 cells, the observed colonies showed smaller dimensions and seemed to be formed by multi-stratified cells, a pattern probably induced by the above-mentioned difficulty in substrate adhesion shown by HT-29 cells when the medium is supplemented with lactate. The colorimetric detection of colonies showed a small, not statistically significant increase in lactate-exposed cells and a statistically significant reduction of the clonogenic power vs the control and vs lactate-exposed cells when BC11 was administered at 20 µM.

## 4. Discussion

Because of its relevance in a large variety of neoplastic diseases, EGFR has long been considered as an attractive therapeutic target [7,55,56]. In cancer cells, EGFR can be abnormally activated by various mechanisms: receptor overexpression, mutations, ligand-dependent receptor dimerization, ligand-independent activation. Usually, these mechanisms lead to the activation of the intracellular tyrosine kinase domain of the receptor. The consequent autophosphorylation of this domain initiates a cascade of downstream signaling pathways involved in the regulation of cellular proliferation, differentiation and survival.

In recent decades, the introduction of EGFR tyrosine kinase inhibitors has produced remarkable clinical results in different cancer forms [57]. Unfortunately, most patients were found to acquire drug resistance over the years and, for this reason, second and third generation inhibitors have been introduced in clinics [58,59]. In spite of these efforts, drug resistance caused by mutations in the EGFR gene or in components of the signal transduction pathways continues to emerge and, recently, novel fourth generation inhibitors have been developed [60,61].

One of the best characterized effects of EGFR-mediated signaling is the activation of glycolytic metabolism [8,62], leading to increased lactate production. As explained in the Introduction, lactate is a signaling molecule, involved in the regulation of gene expression. The experiments described in the present manuscript suggest that enhanced lactate levels can not only be the consequence of the activated EGFR-mediated signaling, but also take an active part in fostering the activated receptor state, generating a vicious feedback loop.

A direct method to dampen this self-sustaining deleterious loop could be the inhibition of LDH-A, with consequent block of lactate production. In several experimental settings, LDH-A inhibition appeared to cause inhibition of EGFR-mediated signaling [63] and, recently, it was also shown to abrogate cancer cell resistance to EGFR tyrosine kinase inhibitors [64]. Unfortunately, although several potential LDH-A inhibitors have been identified in recent years, to our knowledge none of them have been approved for clinical use [65,66].

Our results propose an alternative approach to inhibit EGFR-mediated signaling, which could be considered for those cancer conditions in which receptor activation is mainly triggered by HBEGF. HBEGF is initially synthesized as a transmembrane precursor protein and is then cleaved at the cell surface by the proteases of the ADAM family or by other metalloproteases. In some contexts, the activity of these proteases was found to be promoted by inflammatory cytokines [67] or by a cellular stress response [68].

Soluble HBEGF was found to be implicated in the proliferative potential of tumor cells, contributing to tumor aggressiveness, local invasion, metastasis and chemoresistance [24]. Its increased expression, compared to normal cells, was detected in a number of neoplastic diseases usually characterized by dismal prognosis and/or poor treatment response, such as pancreatic, liver, ovarian and gastric cancers, and glioblastoma [21,22,69,70,71,72].

CRM197 is a nontoxic mutant of diphtheria toxin which binds to human HBEGF and hinders its mitogenic activity [49]. Since it was found to be ineffective in hindering the effects of other EGFR ligands, it is considered as a specific inhibitor of HBEGF [69,73]. At present, CRM197 is approved for clinical use as a carrier protein in multiple conjugate vaccines [74,75]. The antineoplastic activity of CRM197 was investigated in a number of studies, which showed the potential of this HBEGF inhibitor, mainly in increasing the activity of commonly used anticancer drugs, such as cisplatin, doxorubicin and paclitaxel, in cultured human cancer cells from different neoplastic conditions [76,77]; in some models, reversion of drug resistance was also observed [78,79]. Interesting results also came from animal models [80] but, to our knowledge, clinical experience with CRM197 was not encouraging [81]. Remarkably, the difficulties in targeting the EGFR-mediated signaling in cancer have been analyzed in [82].

Our data substantially confirmed the limited anticancer potential of CRM197 when administered as a single treatment (Figure 5 and Figure 6). However, the experimental exposure to increased lactate levels, allowed us to identify the protease playing a major role in releasing HBEGF in our cell system. Our experimental results suggest that the association of CRM197 with an inhibitor of the protease primarily involved in HBEGF release is endowed with anticancer potential, since it can lead to the shutdown of EGFR-mediated signaling and apoptosis. In agreement with these data, it also dramatically reduced cell clonogenic potential and infiltrative growth. Interestingly, this anticancer potential became evident without the administration of chemotherapeutic drugs. Our experiments also evidenced that a single, but sustained exposition to CRM197 can affect cell biology, inducing changes suggesting the reversion of the mesenchymal phenotype; to our knowledge, this effect has never described been so far and deserves further investigation.

## 5. Conclusions

The observations reported here show that, at the level reached in the microenvironment of neoplastic tissues, lactate can induce increased levels of uPA, a protease causing HBEGF shedding and EGFR pathway activation. In different neoplastic contexts, other proteases can be involved in HBEGF shedding [67,68]. Protease inhibitors are already under consideration as possible candidates for anticancer treatment [83]; these compounds have also shown potential in tackling EGFR signaling [84] and increasing the anticancer activity of chemotherapeutic drugs [85]. The observed antineoplastic effect obtained by the association of CRM197 with the uPA inhibitor suggests the possibility of dampening the EGFR-mediated signaling by adopting a tailored treatment, based on the association of CRM197 with a context-appropriate protease inhibitor. In HBEGF-dependent cancers this alternative approach could be considered to overcome the drug resistance frequently observed following the use of conventional EGFR tyrosine kinase inhibitors.

## Figures and Tables

**Table 1:** List of oligonucleotide primer pairs used in the RT–PCR experiments.

Gene	KiCqStart^®^ ID	ID RefSeq	Exons
*MMP2*	H_MMP2_2	NM_004530	11–13
*MMP9*	H_MMP9_1	NM_004994	9–11
*PLAU* (uPA)	H_PLAU_1	NM_001145031	7–8
*LDHB*	H_LDHB_1	NM_001174097	7–8
*LDHA*	H_LDHA_1	NM_001135239	6–7
*ESRRA* (ERR-alpha)	H_ESRRA_2	NM_004451	6–7
*GPER* (GPER1)	H_GPER_1	NM_001039966	2–3
*SRC*	H_SRC_2	NM_005417	5–6
*EGFR*	H_EGFR_3	NM_005228	2–3
*HBEGF*	H_HBEGF_1	NM_001945	4–5
*B2M*	H_B2M_1	NM_004048	2–3
*CYP33* (PPIE)	H_PPIE_1	NM_006112	7–8
*RPS13*	H_RPS13_2	NM_001017	3–4
*TUBA*	H_TUBA1A_2	NM_006009	2–3

**Table 2:** List of the antibodies used for protein detection.

Experiment	Antibody	Host Species	Catalog Number	Producer	Dilution	Time
IB, primary	uPA	rabbit	A2181	AB Clonal ^(a)^	1:2000	2 h
	GPER1	rabbit	A10217	AB Clonal	1:1000	1 h
	ERR-alpha	rabbit	A14184	AB Clonal	1:2000	1 h
	EGFR	rabbit	CPA4394	Cohesion Biosciences ^(b)^	1:1000	1 h
	Phospho-EGFR (Tyr1068)	rabbit	AP0301	AB Clonal	1:1000	1 h
	ERK1/2	rabbit	9102	Cell Signaling ^(c)^	1:1000	16 h (4 °C)
	Phospho-ERK1/2 (Thr202/Tyr204)	rabbit	4370	Cell Signaling	1:1000	16 h (4 °C)
	PUMA α/β	mouse	sc-374223	Santa Cruz ^(d)^	1:500	16 h (4 °C)
	Actin	rabbit	A2066	Merck	1:1000	1 h
IB, secondary	Rabbit IgG Cy5-labeled	goat	111-175-144	Jackson Immuno Research ^(e)^	1:2500	1 h
	Mouse IgG AlexaFluor 647-labeled	donkey	715-605-151	Jackson Immuno Research	1:1000	1 h
IF, primary	E-CAD	mouse	MAB8138	R&D System ^(f)^	1:100	16 h (4 °C)
IF, secondary	Mouse poly-Ig FITC-labeled	goat	F1010	Merck	1:400	30 min

(a): AB clonal: German GmbH, Düsseldorf, Germany. (b): Cohesion Biosciences: London E14 2DN, UK. (c): Cell Signaling: Danvers, MA, USA. (d): Santa Cruz Biotechnology Inc., Dallas, TX, USA. (e): Jackson Immuno Research: Cambridgeshire CB7 4EX, UK. (f): R&D Systems Inc., Minneapolis, MN, USA. IB: Immunoblotting; IF: immunofluorescence. Membranes’ fluorescence was assessed with the Pharos FX^TM^ Scanner (Bio-Rad) at a resolution of 100 µm and bands’ intensities were evaluated using the ImageJ software (version 1.53a).

## Data Availability

The original contributions presented in the study are included in the article/Supplementary Material; further inquiries can be directed to the corresponding author.

## Supplementary Materials

The following supporting information can be downloaded at: https://www.mdpi.com/article/10.3390/cells13181533/s1, Figure S1: Wound-healing assay in HT-29 cultures; Figure S2: Clonogenic assay in HT-29 cultures; Video S1: Wound-healing assay in MDA-MB-231 cultures.
