# Supplementary figures and images for: Lactate-Induced HBEGF Shedding and EGFR Activation: Paving the Way to a New Anticancer Therapeutic Opportunity
Source: Cells. 2024 Sep 13;13(18):1533. doi: 10.3390/cells13181533 (PMC11430493; doi:10.3390/cells13181533)

# Clonogenic assay performed on HT-29 cultures

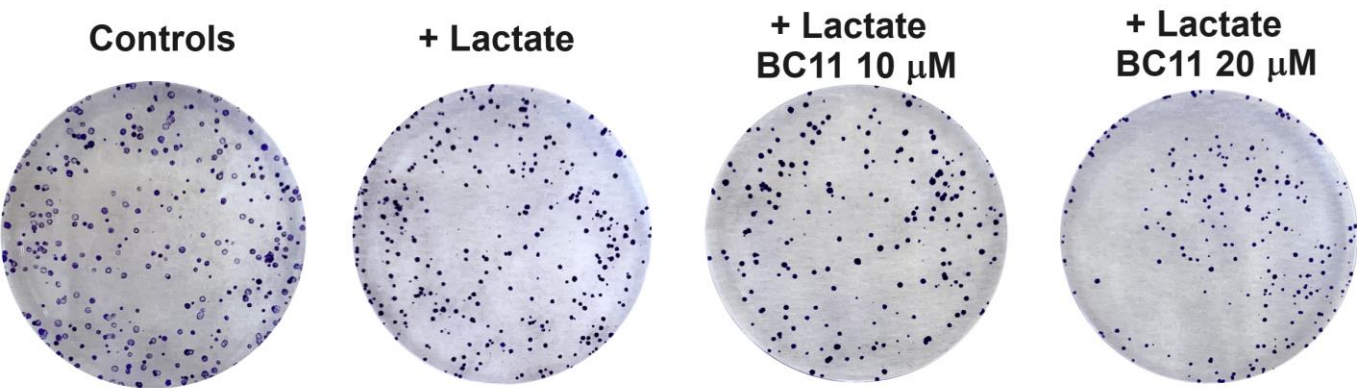

Colorimetric evaluation of colonies

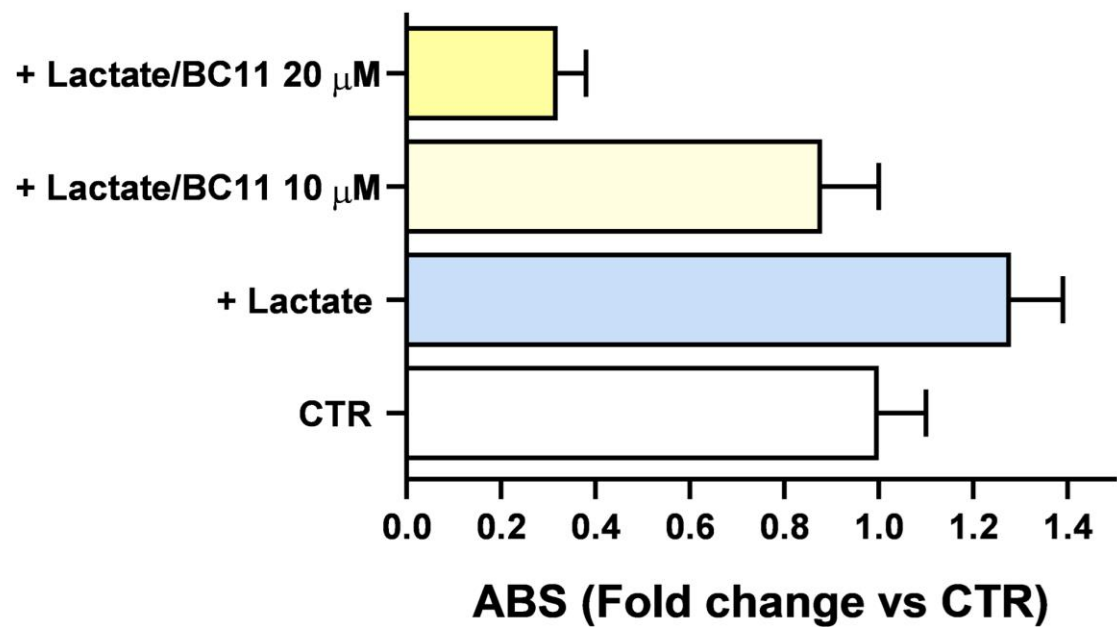

Supplement: Supplementary file 1 [file cells-13-01533-s001.zip › Figure S2.pdf]
